# Accurate Prediction of Mechanical Property of Organic Crystals Using Molecular Dynamics-Based Nanoindentation Simulations

**DOI:** 10.1021/jacs.5c09944

**Published:** 2025-10-03

**Authors:** Sara M. Elgengehi, Durga Prasad Karothu, Weiwei He, Rabindranath Paul, Serdal Kirmizialtin, Panče Naumov

**Affiliations:** † Center for Smart Engineering Materials, 167632 New York University Abu Dhabi, PO Box, 129188 Abu Dhabi, United Arab Emirates; ‡ Program in Chemistry, Division of Science and Mathematics, New York University Abu Dhabi, PO Box, 129188 Abu Dhabi, United Arab Emirates; § Department of Chemistry, 5894 New York University, New York, New York 10003, United States; ∥ Smart Materials Lab, New York University Abu Dhabi, P.O. Box, 129188 Abu Dhabi, United Arab Emirates; ⊥ Research Center for Environment and Materials, Macedonian Academy of Sciences and Arts, Bul. Krste Misirkov 2, MK-1000 Skopje, Macedonia; # Molecular Design Institute, Department of Chemistry, New York University, 100 Washington Square East, New York, New York 10003, United States

## Abstract

Developing reliable methods to predict the mechanical response of organic crystals is essential due to their growing recognition as soft, compliant, lightweight, and ultrafast smart dynamic materials that have applications ranging from pharmaceuticals to biomaterials. Traditional experimental methods, such as nanoindentation, come with practical convenience such as simplicity and efficacy; however, they are restrictive with regard to the surface quality and are subject to other experimental constraints such as the availability of suitable samples and accessibility of as-grown crystal faces. Computational approaches, including quantum mechanics and classical molecular dynamics (MD), offer an alternative approach that is independent of the sample, yet they are known to vary greatly in their predictive accuracy. To account for this shortfall of predictive tools, in this study, we systematically benchmark three major computational methods, including density functional theory (DFT), MD-based approaches based on deformation, and direct nanoindentation simulations, against experimental Young’s moduli. Our results show that the MD-simulated process of indentation conforms most favorably with the experiments and has the lowest mean absolute error. The intermolecular interactions, crystal size, indenter spacing, and unloading rates are identified as the key factors that determine the stiffness, as given by the modulus. Our findings underscore the importance of incorporating both thermal effects and experimental conditions into computational models for improved predictive accuracy and highlight the potential of nanoindentation simulations as a reliable tool for the assessment of the mechanical properties and, possibly, also for the design of organic crystals with predetermined mechanical properties and dynamic behavior.

## Graphical abstract

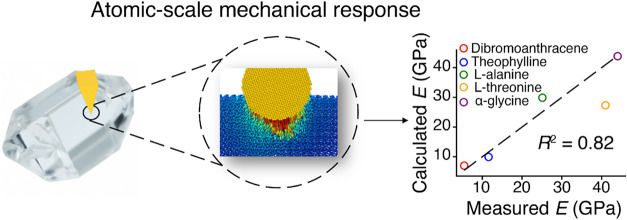

## 1 Introduction

The measurement and understanding of mechanical properties of crystalline materials are becoming indispensable across many fields, including inorganic materials, composites, pharmaceuticals, solid organic materials, and biomaterials,^1–6^ where they remain a bottleneck in the process of implementation of these materials. Owing to the combination of two seemingly contradictory attributes—mechanical softness and crystallinity—this aspect becomes even more relevant in the case of the stability of molecular crystalline solids, particularly in view of their many applications that range from pharmaceuticals, to optical waveguides^7,8^ and actuators,^9,10^ to energetic materials.^11–15^ Indeed, a comprehensive understanding of the mechanical properties of crystalline materials, such as strength, elasticity, ductility, toughness, hardness, and fatigue resistance, provides crucial insights into their suitability for specific application. While various methods are available to assess the mechanical properties, including tensile, compression, flexural, impact, fatigue, creep testing, dynamic mechanical analysis, ultrasonic resonance, and acoustic emission, in practice, the Young’s (“elastic”) modulus (*E*) and hardness (*H*) are among the most commonly used attributes, and are often measured via the widely available and straightforward method of nanoindentation. The Young’s modulus is also a crucial material property commonly focused on in various computational modeling techniques aimed at both analysis and design of materials. It has been approached with methods such as finite element analysis (FEA), materials selection and design, impact analysis, dynamic analysis, thermal analysis, and fatigue analysis. Alternative methods for the determination of the Young’s modulus include Brillouin scattering, atomic force microscopy coupled to indentation, three/four-point bending, resonant ultrasound spectroscopy, and impulse excitation; however, these techniques are usually demanding in sample size and are not readily accessible. While the nanoindentation compensates for some of these drawbacks, its accuracy depends directly on the availability, accessibility, and quality of specific sample surfaces with minimal roughness. The method also requires great care with statistics based on a large number (hundreds) of measurements to be performed on a chemically stable sample; due to these challenges, while being very convenient, nanoindentation is not always feasible. Moreover, the preparation of crystals of good quality is not always possible; some crystal faces may be underdeveloped or physically inaccessible, and mechanical testing of unstable energetic materials can be subject to safety concerns.

Computational methods emerge as a viable method for modeling the mechanical response of organic crystals, enabling the evaluation of the Young’s modulus even at the limited applicability of experimental methods. They offer practical, efficient, and time- and resource-effective estimates. Quantum mechanics (QM), specifically *ab initio* methods such as density functional theory (DFT) that model crystalline systems at the quantum level, have been utilized to estimate the mechanical properties of organic crystals.^16–18^ While the plain Hartree–Fock method is not predictive for the elastic properties of molecular crystals, the scaled-corrected Hartree–Fock method (s-HF-3c) has been recently demonstrated^19^ to yield elastic tensors comparable in accuracy to dispersion-corrected DFT. However, their applicability is limited to systems containing only a few hundred atoms; as a result, they do not incorporate intergranular boundaries. Furthermore, standard QM-based methods are conducted on minimum-energy structures at zero temperature, overlooking the thermal and anharmonic effects. However, while QM performs well for materials with *E* > 20 GPa, it generally tends to overestimate mechanical properties for softer materials. Molecular dynamics (MD) simulations offer a complementary route by explicitly incorporating finite-size and thermal effects. Brunsteiner et al.^20^ investigated the elastic properties of organic crystals, subjecting them to compression and elongation along the coordinate axes. As an alternative approach, nanoindentation simulations have been advocated as a more direct method to emulate experimental conditions. For example, Chen et al.^21^ examined the indentation dynamics of cyclotrimethylenetrinitramine (RDX), and Mathew et al. applied this approach to the explosive organic crystal 1,3,5-triamino-2,4,6-trinitrobenzene (TATB).^22^ While MD simulations explicitly account for thermal effects and reduce the adverse effects of finite size, the empirical potentials used in MD simulations can introduce systematic errors. QM provides accurate energetics, while MD captures thermal and fine-size effects better, raising the important question of which approach describes the mechanical properties of organic crystals more accurately.

To address these issues, we conducted a comparative study that aims to assess the reliable prediction of the mechanical response of organic crystals as measured by nanoindentation experiments. Here, we examine five organic crystals spanning a range of mechanical properties, from soft to hard, with *E* = 5–50 GPa. Our results show that while overall MD simulations perform better compared to the DFT methods, the nanoindentation simulations are superior to both approaches. Led by this result, we then investigated the factors that contribute to the stiffness of organic crystals and identified the weak forces along the indentation direction to be the major contributors. Beyond the intrinsic structural factors, we establish that while oftentimes overlooked, specific experimental details are in fact critically important. Notably, the values of the estimated Young’s modulus from simulations are sensitive to the unloading rate, the crystal size, and the density of indents. Since the relevance of these experimental factors is often overlooked in routine measurements, our findings emphasize the need to report additional measurement details to ensure transferability of the mechanical properties of organic crystals.

## 2 Results and Discussion

To evaluate the accuracy of computational approaches in predicting the stiffness, expressed as *E*, five crystal structures were selected that represent a range of intermolecular interactions, from weak van der Waals forces to stacking interactions and strong hydrogen bonding. The crystals of 9,10-dibromoanthracene (DBA), theophylline (anhydrate form), l-alanine, l-threonine, and α-glycine were first studied experimentally to obtain their stiffnesses in specified directions. Later, the same materials were subjected to simulations. The Young’s moduli reported here correspond to directional indentation moduli measured or simulated along specific crystallographic faces: (001)/(001̅) for DBA, l-alanine, l-threonine, and α-glycine; (100)/(1̅00) for theophylline. Figure 1a,b illustrates the compounds and nanoindentation model, and Figure 1c shows the time evolution of a typical indentation process where the displacement vectors are monitored during loading and unloading. Simulation systems of 20 × 20 × 20 nm^3^ were constructed, containing 580,608–1,033,600 particles in each system (the large simulation systems were found to be crucial for accurate predictions of *E*, *vide infra*). A spherical nanoindenter made of iron nanoparticles was loaded and unloaded along the indentation direction, with an unloading rate of 0.008 m s^–1^. Although this rate is about 3 orders of magnitude faster than those used in typical nanoindentation experiments, to our knowledge, it is the slowest rate used in simulating organic crystals. The relatively slow rate helps reduce systematic errors caused by the rapid dissipation of energy during unloading relaxation. Similar to the experiments, the resulting force–displacement curves were fitted to the Oliver-Pharr (O–P) method^23,24^ to estimate *E*.

**Figure 1:**
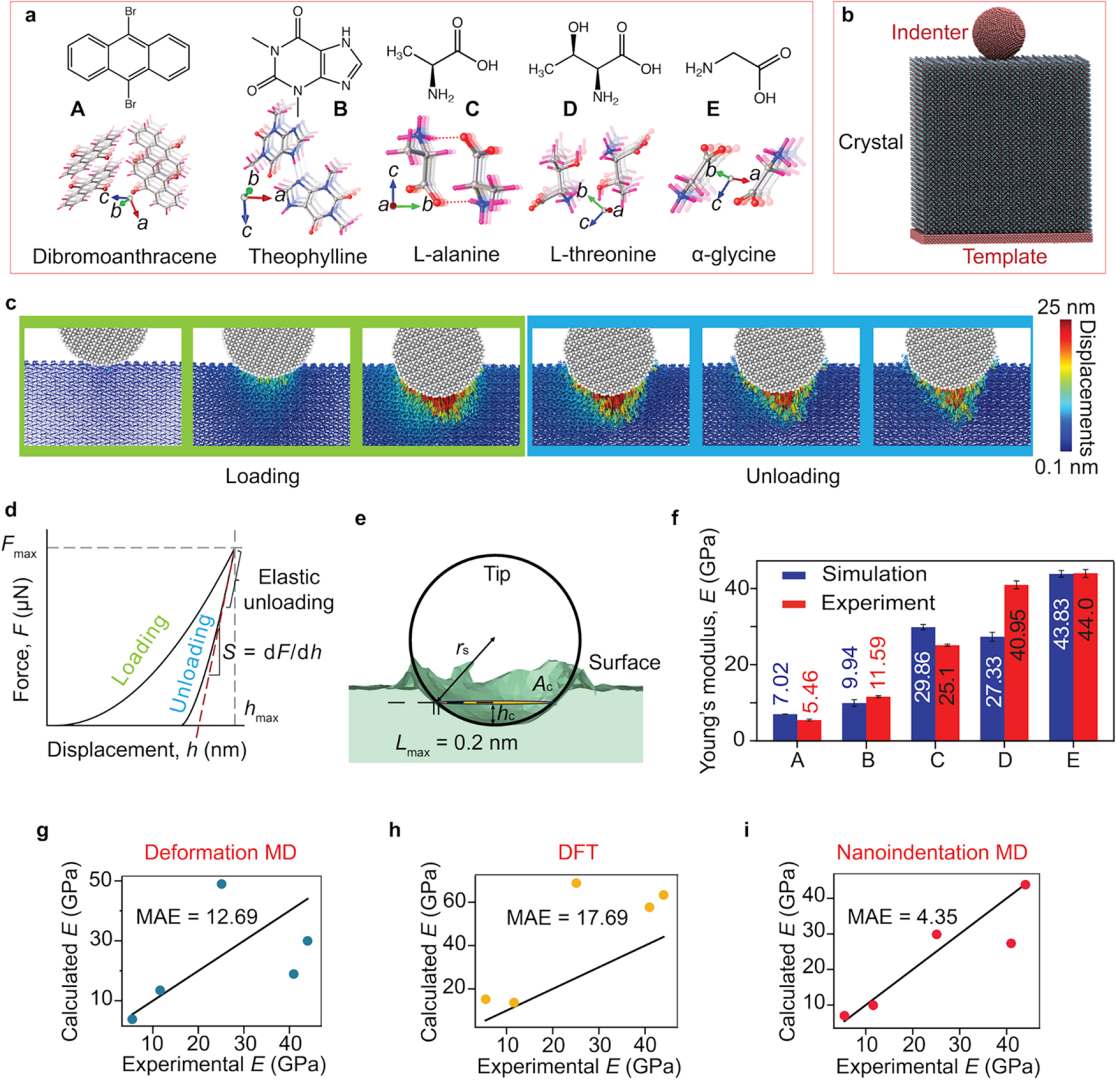
Computer simulations used to assess the calculated Young’s moduli. (a) Chemical structures and crystal packing of five crystal structures studied experimentally and computationally: (A) dibromoanthracene (DBA), (B) theophylline, (C) l-alanine, (D) l-threonine, and (E) α-glycine. (b) Schematic of the nanoindentation simulation setup, showing a crystal, indenter, and a supporting template, mimicking a typical experimental setup. (c) Snapshots of the crystal during loading and unloading, colored by displacement vectors that allow atomic-scale visualization of the process. (d) The force displacement curves obtained during indentation were used in the Oliver-Pharr (O–P) method to estimate Young’s Modulus (*E*). (e) Surface mesh diagram depicting the definitions of contact depth (*h*_c_) and contact area (*A*_c_) used in the O–P method. (f) Comparison of *E* values from nanoindentation simulations with experimental data. The bars represent standard errors derived from three independent simulations. (g–i) Correlation plots of calculated *E* values from different computational methods, with a line added to each graph to guide the eye for ideal agreement between experiment and simulation. The accuracy of each method is quantified by the mean absolute error (MAE): (g) deformation MD, (h) DFT: for DBA and theophylline, we used PBE-TS, while for the other systems we used PBE-TS or PBE-D3,^18,25^ and (i) nanoindentation MD simulations methods. The nanoindentation MD simulation shows the lowest MAE of 4.35, demonstrating the highest accuracy. Error bars are smaller than the plotted symbols and therefore cannot be seen.

### 2.1 Comparison of the Indentation Simulations with Experiments and Other Computational Methods

In order to assess the performance of the present approach, the results were compared with experiments and computer simulations based on DFT methods and deformation. For DFT simulations, we used published results when available; namely, we adopted the elastic constants for l-alanine,^18^ l-threonine,^25^ and α-glycine^18^ from earlier works. These constants were obtained using the PBE-TS functional, with the exception of l-threonine, for which only the PBE-D3 data were used. In the case of DBA and theophylline, we followed a methodology similar to that in ref 18,25 and we used PBE-TS for all our calculations. In order to compare our approach with deformation simulations, we adopted the method of Brunsteiner et al.^20^ We used the same simulation setups as in the nanoindentation simulations to circumvent discrepancies due to differences in the molecular setup and force field parameters. In Figure 1f, we compare our nanoindentation simulations to experimental data. Figure 1g,h, and i show the performance of the three computational methods (the line on each graph guides the eye for perfect agreement between experiment and simulation). To quantify the performance of each method, the mean absolute errors (MAE) of *E* were calculated between the experiment and simulation.

The DFT approach yields an MAE of 17.69 GPa, with systematic overestimation for all crystals studied (Figure 1h). In particular, l-alanine was found to deviate most between experiments and theory. The deformation method, which employs MD simulations, performed better, achieving an overall MAE of 12.69 GPa. While this method reproduces well the values for soft crystals, it deviates significantly from the measured values for hard crystals. The nanoindentation simulations were found to provide the best agreement with an overall MAE value of 4.35, with l-threonine showing the largest deviation. The shared discrepancy of l-threonine between the two MD-based approaches suggests limitations in the fixed-charge empirical potential for this material. Indeed, increasing the charges of the amino group and hydroxyl-group hydrogens by +0.1 and reducing the carboxylate oxygen pairs by −0.1 yield *E* = 37.44 GPa, suggesting that further improvements could be achieved using polarized or machine learning-based force fields. Overall, the results suggest that MD provides better agreement compared with DFT approaches, and among the MD-based approaches, the nanoindentation method aligns better with experimental measurements.

### 2.2 Correlation of the Deformation Dynamics with Stiffness

Nanoindentation simulations that accurately reproduce *E* values also allow for atomic-scale visualization of the indentation process (Figure 2). The energy frameworks along different crystallographic axes offer a visual representation of the directions of intermolecular interactions. The radii of these cylinders are directly proportional to the strength of the corresponding intermolecular interactions, providing a visualization of the energetic framework of the crystal structure.^26,27^ As a general trend, from softer to harder crystals, the edges of the frameworks become wider. The only exception is l-threonine, where the framework is thinner than l-alanine, while the experimental *E* values show relatively higher stiffness for l-threonine. Interestingly, the framework thickness correlates better with the computed *E* values, suggesting consistency in the computational representation of the molecular systems.

**Figure 2:**
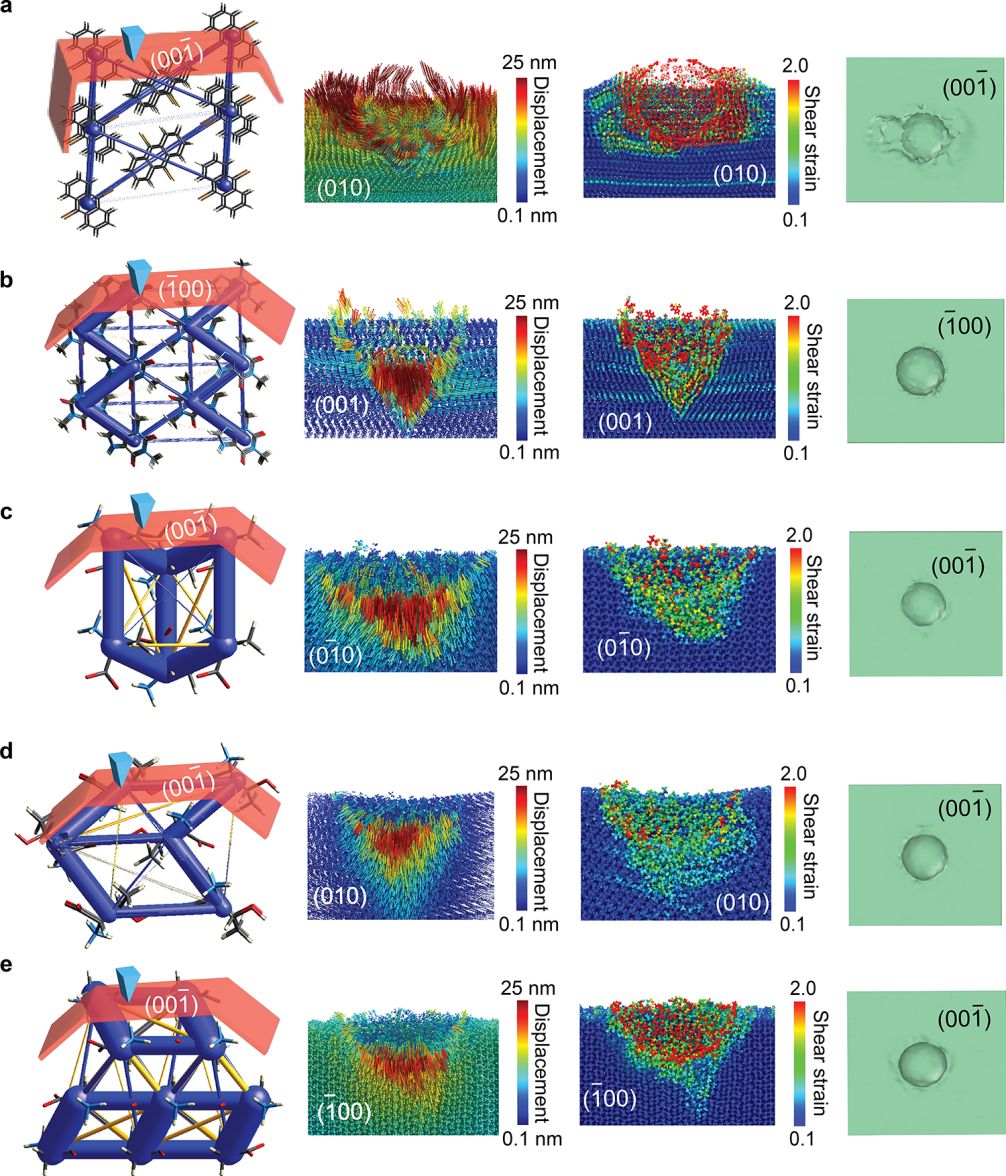
Deformation dynamics of the crystals. Face indices, energy frameworks, displacement vectors, shear strain, and surface meshes are shown for (a) DBA on the (001̅) face, (b) theophylline on the (1̅00) face, (c) l-alanine on the (001̅) face, (d) l-threonine on the (001̅) face, and (e) α-glycine on the (001̅) face. The relative thickness of the energy frameworks (far-left panels) reflects the comparative interaction strengths. The arrows of the displacement vector represent the orientation of each atom due to the surface disruption, with the color-coded scale representing the lowest (blue) and highest (red) displacement and shear strain for each crystal. Based on this, enhanced atomic migrations and surface disruption are observed in the case of soft crystals in general. Note that for the energy frameworks (left panels), the scaling of the tubes in panels a and b is identical, while panels c, d, and e share a different but consistent scaling. The relative thickness of the tubes follows a 1 : 3 ratio between panels a, b and panels c, d, e, reflecting the comparative interaction strengths.

Figure 2 also shows the displacement vectors after loading. The displacements are primarily confined to the layers within the region of maximum displacement along the indentation direction, where we observe differences among the crystals. The surface disruption with enhanced atomic migrations is visible in the case of soft crystals, and the disturbance decreases as the modulus increases. In the crystal of theophylline, some sliding occurs in the layers near the surface during the indentation, which can be attributed to the flat slip planes stabilized by the hydrogen-bonded columns.^28^ The hydrogen bonds here represent relatively weak interactions, making it easier for the layers to slide past one another under applied stress. However, for α-glycine, despite its high modulus, the displacements around the indenter tip are greater than those of l-alanine and l-threonine, highlighting the effect of anisotropy. After complete unloading, the dislocations are reduced for all crystals, except for DBA, as shown in Supporting Information (SI) Figure S1. The most significant recovery of migrations is observed for l-threonine, indicating its ability to recover upon tip unloading. Similarly, atomic sliding in the upper layers of the theophylline crystal exhibits partial recovery after unloading (SI Figure S1).

To further analyze the deformation mechanisms, we computed the degree of distortion between adjacent layers. For that purpose, the von Mises local shear invariant (shear strain) was calculated for each particle based on the deformation gradient tensor. Figure 2 illustrates the nucleation of the deformation at the maximum indentation depth, where deformation concentrates near the indenter’s tip, reflecting the onset of plastic deformation during the indentation process. Interestingly, for the crystals of DBA and theophylline, it was observed that shear bands appear near the upper layers, suggesting that in the case of DBA, the weak van der Waals interactions facilitate localized plasticity, whereas in theophylline, the columnar hydrogen-bonded slip planes contribute to the observed shear bands. As the modulus increases, the strain decreases accordingly, except in the case of α-glycine, as discussed with relation to the displacement vectors (*vide supra*). The displacement vectors and shear strain during the unloading also showed the flow of molecules onto the indenter as the mechanical stress is released (SI Figure S1). The “stickiness” increased as the material softens, consistent with the findings of a previous study by Chen et al.^21^ The surface mesh analysis further aided in visualization of these deformation effects, particularly the residual shape left after loading (Figure 2) and unloading (SI Figure S1), which offer insights into the material’s response to applied stress. Unlike other crystals, DBA exhibits a more pronounced dissipation of mechanical deformation along its surface, indicative of its greater plasticity. Nanoindentation using MD simulations provides atomic-level insights into the mechanical response of organic crystals during loading and unloading, while it also helps identify key factors that determine the modulus. A critical feature is the relative orientation of weak forces and the indentation direction. When these vectors are parallel, the material exhibits greater resistance to the applied force, resulting in a higher *E*. Conversely, when the vectors are perpendicular, the material becomes softer and more susceptible to deformation. Among the factors, hydrogen bonding and stacking interactions along the indentation direction were found to play a crucial role in determining the values of *E*. Incorporating these intermolecular features into a linear regression model yields a strong (91%) correlation with *E* (Figure 3).

**Figure 3:**
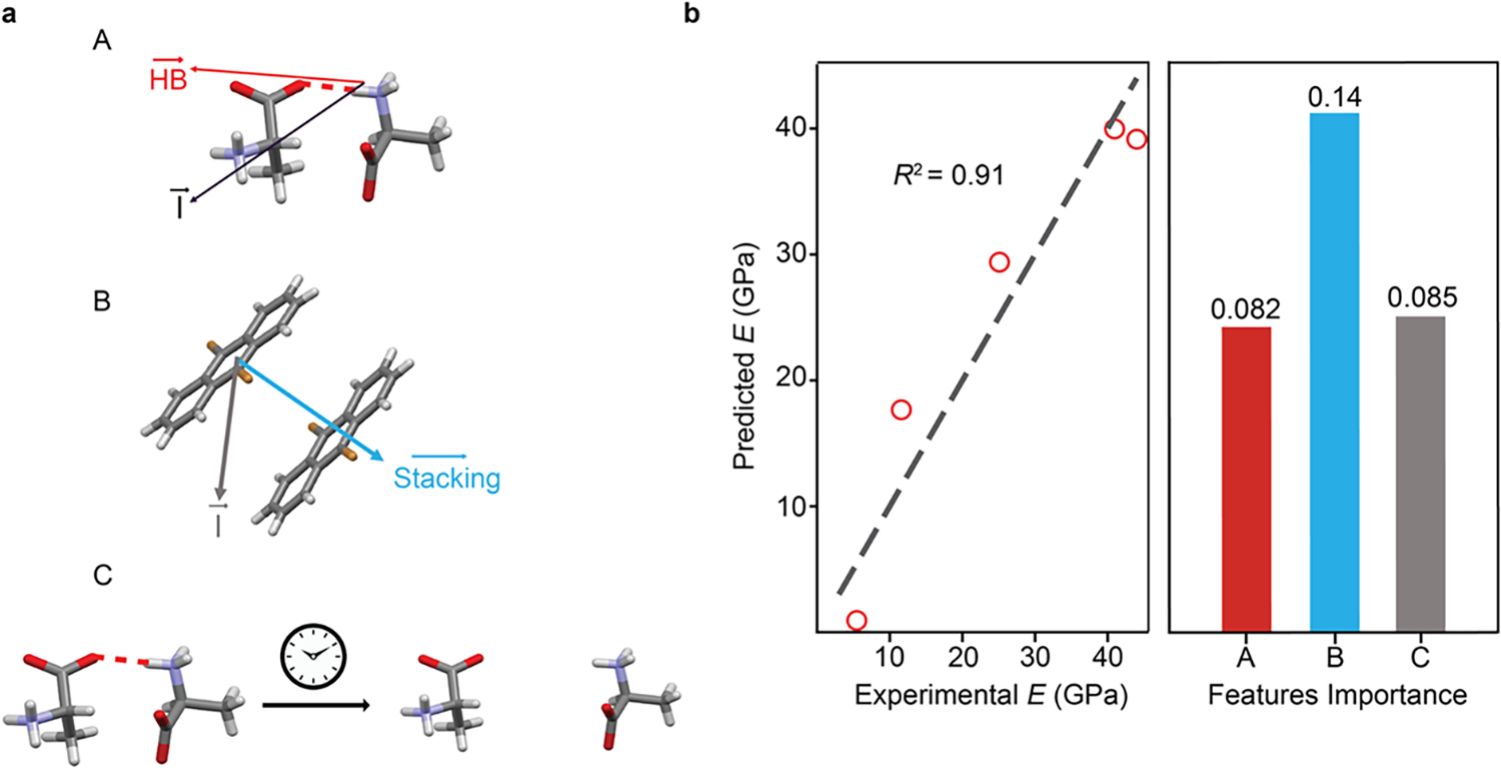
Linear regression model correlating Young’s moduli to molecular interactions. (a) The intermolecular forces are classified as hydrogen bonding and base stacking terms, with the strength of hydrogen bonding estimated from hydrogen bonding residence times. HB, “stacking”, and I here stand for the hydrogen bonding, stacking, and indentation directions, respectively. The model incorporates forces along the indentation direction to estimate *E*. (b) Linear regression model based on these three terms fit the experimental values of *E* as *E* = 151.4–31252.9x_*A*_ – 561637.2x_*B*_ – 8.26x_*C*_, where x_*A,C*_ are the values of the factors computed from simulation data. Feature importance analysis gives a relatively higher emphasis to stacking.

### 2.3 Dependence of the Young’s Modulus on Crystal Size, Unloading Rate, and Indent Density

The success of nanoindentation simulations in reproducing the experimental results motivated researchers to explore the factors contributing to the modulus. In addition to the molecular structure and packing, the parameters of the experimental setup are empirically known to affect the reported values. Computer simulations provide a controlled environment where individual parameters can be systematically varied while others are kept constant, thereby enabling a rigorous assessment of the experimental parameters.

First, the effect of the crystal size on the mechanical properties was studied on l-alanine by varying the depth of the crystal (Figure 4a). Subsequently, the effect of the crystal surface was assessed by increasing the area orthogonal to the indentation direction. Then, the overall crystal size was assessed by doubling the crystal in all directions. As a second experimental parameter, the effect of the unloading rate was investigated (Figure 4b). The indenter was retracted at unloading rates: 0.8, 0.4, 0.08, and 0.008 m s^–1^, respectively. Lastly, the effect of the indenter spacing was investigated. Since indenter arrays are commonly used to measure the elastic modulus, the effect of indenter spacing on *E* was studied. Here, “indenter spacing” refers to the ratio of the unit cell dimension to the indenter diameter (Figure 4c). Therefore, smaller spacing values correspond to larger indenters, which produce a broader stress distribution. This setting allows us to mimic different indenter arrays in an experimental setup. To examine this effect, the crystal size was fixed at approximately 20 × 20 × 20 nm^3^, and the diameter of the indenter was varied from 4 to 12 nm in a periodic simulation system.

**Figure 4:**
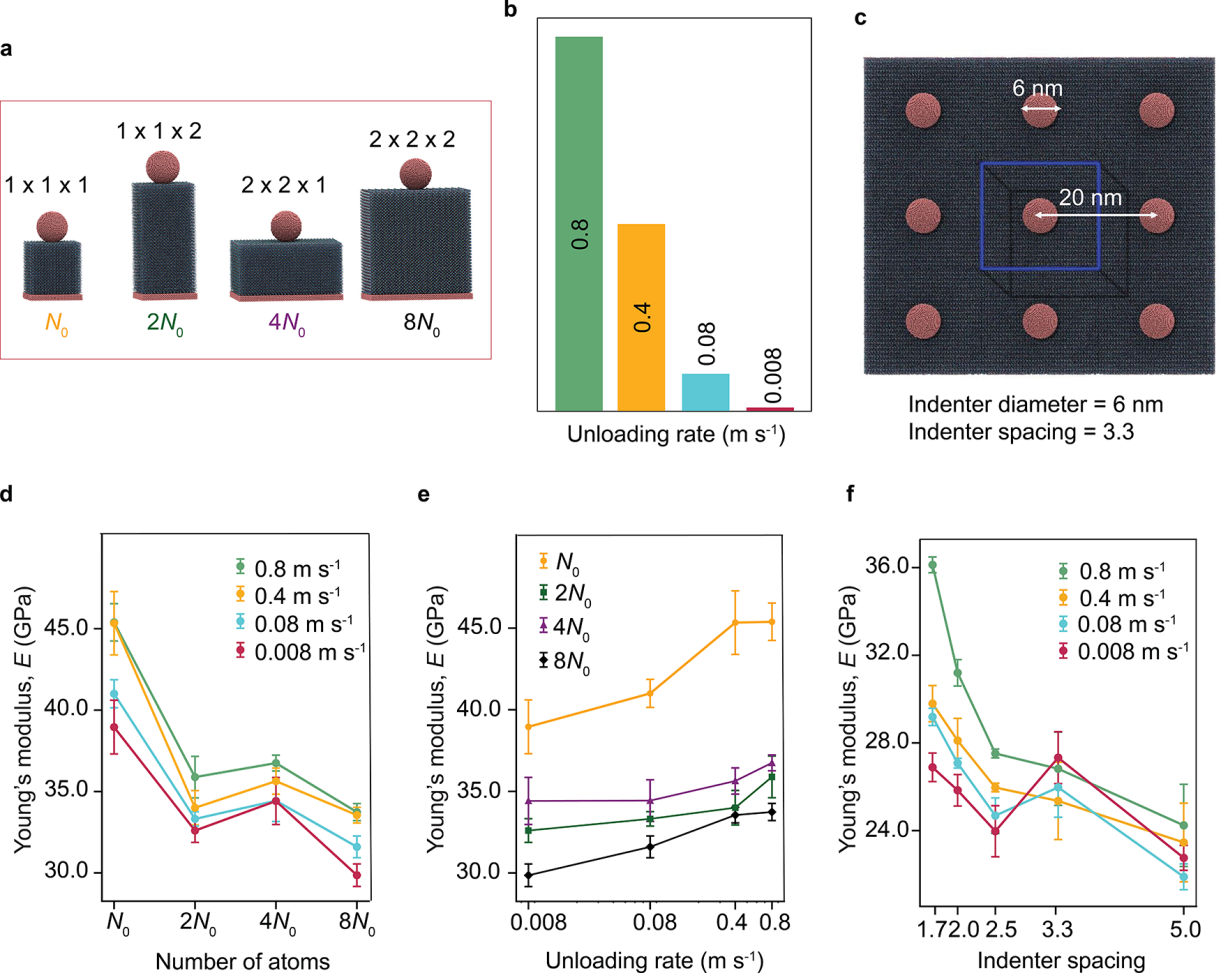
Factors affecting Young’s modulus. (a) Crystal sizes studied: *N*_0_ the smallest crystal size ∼10 × 10 × 10 nm^3^ (*x* × *y* × *z*, 120224 atoms), 2*N*_0_ (*x* × *y* × 2*z*, 240448 atoms), 4*N*_0_ (2*x* × 2*y* × *z*, 480896 atoms), and 8*N*_0_ (2*x* × 2*y* × 2*z*, 961792 atoms). Here, *N*_0_ represents the minimum number of particles within the smallest crystal size. (b) Different unloading rates of indentation, starting from 0.8 to 0.008 m s^–1^. (c) A diagram showing the indenter spacing calculated by dividing the distance between the unit cell and its image by the indenter diameter. (d) The effect of size on the value of the Young’s modulus, *E*. The modulus decreases as the size increases at different rates. (e) Effect of changing unloading rates on *E*, showing a decrease in *E* as a function of lowering the unloading rates. (f) The indenter spacing affects the modulus, demonstrating an almost linear correlation between the spacing and the reported values of *E*. These trends remain the same for unloading rates. The error bars show the standard errors estimated by running three independent simulations for each data point.

The effect of these parameters on the *E* values is shown in Figure 4d–f. The simulation results indicate that larger crystals tend to exhibit softer mechanical behavior compared with smaller crystals. This suggests that larger crystals allow for a greater distribution of stress and the development of the plastic zone, while smaller crystals may introduce boundary effects that distort the deformation patterns.^22,29^ This conclusion aligns with conclusions from AFM nanoindentation studies, where millimeter-sized cocrystals were found to be 40% softer than nanometer-sized ones.^30^ This trend remains consistent across different unloading rates. We note that although complete convergence with system size was not achieved, the change from 2*N*_0_ to 8*N*_0_ is ∼5%, and further increases of 4× would likely alter the modulus by only ∼2% and thus within the uncertainty limits of our approach. Similar to crystal size, the unloading rate also impacts the material’s response. In simulations, the estimated Young’s modulus shows sensitivity to unloading rate because higher rates leave insufficient time for atomic relaxation beneath the indenter, producing an apparent hardening effect.^31,32^ This variation of the modulus does not reflect a change in the intrinsic property of the material, which remains rate-independent; rather, it highlights known artifacts in the measurement of these properties. As the unloading rate decreases, the Young’s modulus also decreases because the atoms beneath the indenter have more time to readjust their positions (Figure 4e). Lastly, the calculated moduli at different indenter spacings also show systematic variation (Figure 4f): the modulus increases with decreasing indenter spacing. This trend persists across different unloading rates, indicating that higher applied pressure reduces the material’s plasticity.^31,32^

While computational results vary with setup, experimental measurements are also sensitive to factors such as sample preparation, alignment, environmental conditions, and data interpretation, making direct comparison and perfect agreement with “ideal” bulk properties challenging. Computational methods, though based on idealized models, can reproduce experimental trends and sometimes benefit from error cancellation when compared with measured values. While some cases in the literature show good agreement between experiment and theory, others exhibit little to no correlation due to these complexities. This underscores the need for a careful experimental design and execution to obtain precise values. In our view, the most reliable path toward determining true intrinsic material properties lies in a combined, iterative approach that integrates theoretical predictions with experimental measurements, allowing systematic deviations to be identified and results to be refined toward reflecting the true material behavior.

## 3 Conclusions

This study demonstrates that molecular dynamics (MD) simulations can reliably predict the stiffness of organic crystals, expressed as the Young’s modulus. Compared with density functional theory (DFT) and conventional deformation-based MD methods, nanoindentation simulations exhibit the best agreement with experimental measurements for organic materials across various ranges of elasticity. We also note that refining empirical potentials to incorporate polarization effects could further improve the accuracy of the predictions. By replicating the essential features of the nanoindentation process, our approach provides atomic-level insights into the mechanical response of organic crystals. Simulations reveal that soft crystals exhibit intense shear strain and plastic deformation, whereas harder crystals absorb stress and partially recover upon unloading. Hydrogen bonding and stacking interactions along the indentation direction were found to be the key structural and energetic components that determine the stiffness. Although the intermolecular interactions play an important role in determining the modulus, we show that the experimental measurement conditions, such as crystal size, unloading rate, and indenter spacing, which are often overlooked, are also major contributors to the measured properties. The crystal size determines the stress distribution and plastic zone formation, while the unloading rate impacts the extent of elastic recovery.^32^ Additionally, the indenter spacing, controlled by the indenter templates, plays a significant role; smaller indenter spacing leads to higher moduli due to greater applied pressure and reduced material plasticity, in line with earlier observations.^31,32^ This study emphasizes the need for careful consideration of experimental parameters when comparing computational and experimental results and advocates for standardizing computational and experimental methodologies to enhance reproducibility and comparability across studies. Beyond improving the estimated moduli, this work uncovers the broader potential of nanoindentation MD simulations for understanding the mechanical behavior of ordered solid matter. The ability to correlate structural and energetic properties with mechanical responses enables a framework for designing materials with specific mechanical properties.

## 4 Methods

### 4.1 Experimental Methodology

#### 4.1.1 Materials

l-Threonine, α-glycine, l-alanine, and 9,10-dibromoanthracene (DBA) were purchased from Sigma-Aldrich. Commercially available solvents were used for crystallization as received, without further purification.

#### 4.1.2 Single-Crystal X-ray Diffraction

Single-crystal X-ray diffraction data for l-threonine, α-glycine, l-alanine, and DBA were collected using a Bruker DUO diffractometer equipped with microfocus Mo*K*_α_ radiation (*λ* = 0.71073 Å) and a Photon II detector at selected temperatures. The unit cell parameters were determined, and data collection was performed using the APEX3 program.^33^ The diffraction frames were integrated with the Bruker SAINT software,^34^ and absorption corrections were applied using the SADABS program.^35^ Structural determination and refinement were conducted through the OLEX2 interface,^36^ employing the full-matrix least-squares method based on *F* ^2^ against all reflections via SHELXL-2014/7.^37^ The final CIF was examined for overlooked symmetry elements using the program PLATON.^38^ The figures showing the crystal packing were generated with OLEX2 ^36^ and Mercury.^39^

#### 4.1.3 Nanoindentation Measurements

Prior to nanoindentation experiments, the unit cell parameters and crystal faces of manually selected crystals were determined by using single-crystal X-ray diffraction and a face indexing software.^33^ The nanoindentation was performed using an Agilent G200 nanoindenter equipped with an XP head and a Berkovich diamond indenter. The continuous stiffness measurement technique was utilized, maintaining a strain rate of 0.05 s^–1^, an oscillation amplitude of 2 nm, and a frequency of 45 Hz. The indentation was conducted at selected depths on accessible crystallographic faces. Before testing, the stiffness and geometry of the indenter were calibrated by using a Corning 7980 silica reference sample (Nanomechanics S1495–25). A Poisson’s ratio of 0.30 was assumed in the mechanical property calculations. The load–displacement data obtained from the experiments were analyzed using the Oliver-Pharr method^23,40^ to determine mechanical properties and the projected contact area of the crystal. The values of *E* for some of the compounds were extracted from previously published reports,^17,41^ and confirmed experimentally.

### 4.2 Computational Methodology

#### 4.2.1 Simulation Setup

A simulation box was constructed orthogonal to the indentation directions with varying dimensions, as shown in Figure 1b (for details, see the Results and Discussion section). A vacuum slice was introduced by extending the simulation box along the indentation direction to accommodate the indenter and prevent interactions between the periodic crystal layers. To mimic the experimental indentation setup, an iron template was positioned below the crystal to keep the bottom layers of the crystal fixed during the indentation. An iron spherical indenter was introduced above each indentation face. Although a pyramidal Berkovich indenter is typically used in experiments, its rounded tip of radius of ∼150 nm means that at the nanometer scale, contact effectively occurs with an approximately round tip. This makes the spherical indenter in simulations a suitable approximation for capturing the relevant contact mechanics, as it has been assumed in previous studies.^22,31^ The AMBER force field^42^ was used to model the interactions of the organic crystal structures with the partial charges fitted to the B3LYP/6–311g­(d,p) level of theory.^43^ Additional details on the preparation and simulation setup are summarized in the Supporting Information.

#### 4.2.2 Nanoindentation Simulation Protocol

The indentation simulations were performed by steered molecular dynamics, where a force vector was applied to an indenter using the GROMACS^44^ software in conjunction with the PLUMED plugin.^45^ During the simulation, the indenter was advanced into the crystal in a displacement-controlled manner at a penetration rate of 8.3 m s^–1^. An equilibration stage of 1 ns was provided to allow the dislocations to equilibrate and decrease significantly, and then the indenter was retracted using varying unloading rates. Analysis of the atomic-scale deformation parameters was conducted using the Open Visualization Tool (OVITO)^46^ software package. The force–displacement data was analyzed based on the Oliver–Pharr (O–P) method^23,24^ to determine the Young’s modulus of each organic crystal, which required estimation of the contact area by using the same method. A schematic representation of the contact area *A*_c_ and the depth *h*_c_ between the tip and the crystal is shown in Figure 1e. Additional details on the simulation and analysis protocol are provided in the Supporting Information.

#### 4.2.3 Density Functional Theory (DFT) Calculation of the Elastic Tensors

DFT calculations were performed on single unit crystals. Geometry optimizations were performed first by minimizing both the atomic positions and the lattice parameters. All calculations were carried out using CASTEP,^47^ which employs the DFT plane-wave pseudopotential method. The Perdew–Burke–Ernzerh (PBE) of generalized gradient approximation functional (GGA)^48^ was used with ultrasoft pseudopotentials.^49^ Pairwise dispersion-interaction contributions were considered using the Tkatchenko-Scheer (TS) method.^50^ The plane-wave basis set cutoff energy was chosen at the ultrafine level of accuracy. For all calculations, the Monkhorst–Pack grid was employed to sample the Brillouin zone, using a fine k-point mesh with a maximum spacing of 0.07 Å^–1^. The Broyden–Fletcher–Goldfarb–Shanno (BFGS) algorithm was adopted during energy minimizations to ensure a reliable optimized structure.^51^ The structure was considered to have converged when the free energy value reached below 1 × 10^–5^ eV/atom, the maximum force below 0.03 eV/Å and the maximum displacement below 0.001 Å. All CASTEP input files were generated using Materials Studio^52^ and run in a standalone mode.

The optimized crystal structures were then used as an input to calculate the full tensor of second-order elastic constants. A series of distorted structures was generated using Materials Studio^52^ and then processed in standalone mode for geometry optimization, with the lattice parameters of each distorted structure kept fixed. To ensure accurate calculation of the stiffness tensor, the convergence criteria were changed to fine settings with thresholds of 2 × 10^–6^ eV/atom for the free energy values, 0.006 eV/Å for the maximum force, and 0.0002 Å for the maximum displacement. The plane-wave basis set energy cutoff was maintained identical to that used in the initial geometry optimizations. The SCF convergence criteria were set to 1 × 10^–6^ eV/atom in all calculations. The elastic-constants script^53^ was used as an alternative to Materials Studio to obtain the full 6 × 6 elastic tensors. These tensors were then used as input for MechaPredict^18^ to calculate the face-specific Young’s moduli of the crystals to compare directly with the nanoindentation simulation and experimental results. MechaPredict projects the calculated stiffness tensors along the user-specified crystallographic face.

#### 4.2.4 Strain Analysis from MD Simulations

To evaluate the mechanical properties from molecular dynamics simulations, the GROMACS^44^ software suite was employed. The gmxstrain tool,^20^ was utilized to determine the elastic properties of the organic crystals. The force field was chosen to be the same as the nanoindentation simulation (described above). Each supercell was then subjected to a series of uniaxial and shear deformations, with strain values of 0.004, 0.008, and 0.012, selected to remain within the elastic regime while maintaining numerical stability. The simulations were performed at a constant number of particles, volume, and temperature (NVT), where the stress response to the applied strain was averaged over the simulation time. The components of the stiffness tensor were then computed from the stress–strain relationships, following the generalized Hooke’s law.^54,55^ Using the calculated stiffness tensor and system parameters from the MD, MechaPredict^18^ was used to obtain the *E*_*hkl*_ for the studied crystals.

#### 4.2.5 Energy Frameworks Analysis

The intermolecular interaction energies were derived from the B3LYP/6–31G­(d,p) wave functions and partitioned into the electrostatic (*E*_elec_), polarization (*E*_pol_), dispersion (*E*_disp_), and repulsion (*E*_rep_) components. CrystalExplorer 17 ^26,27^ was employed to construct the energy frameworks.

## Supplementary Material





## Notes

Computational methods for molecular modeling, nanoindentation MD setup, and Young’s modulus evaluation; displacement vectors, shear strain, and surface mesh after unloading (Figure S1) (PDF)
MD nanoindentation input/output files for DBA, theophylline anhydrate, l-alanine, l-threonine, and α-glycine, PLUMED control file, Python script for Oliver–Pharr fitting (calculation_of_E.py) (ZIP); DFT input/output files and parameters for optimization calculations (ZIP)
