# Supplementary material for: Accurate Prediction of Mechanical Property of Organic Crystals Using Molecular Dynamics-Based Nanoindentation Simulations
Source: J Am Chem Soc. 2025 Oct 3;147(41):37186–95. doi: 10.1021/jacs.5c09944 (PMC12532296; doi:10.1021/jacs.5c09944)
Supplement: Supplementary file 1 [file ja5c09944_si_001.pdf]

Supplementary Information

Accurate Prediction of Mechanical Property of Organic Crystals Using Molecular Dynamics-Based Nanoindentation Simulations

Sara M. Elgengehi[1,3], Durga Prasad Karothu[1*], Weiwei He[2], Rabindranath Paul[1], Serdal Kirmizialtin[1,2,3*], Pance Naumov[1,2,4,5,6*]

[1]*Center for Smart Engineering Materials, New York University Abu Dhabi, PO Box, 129188 Abu Dhabi, United Arab Emirates*
[2]*Program in Chemistry, Division of Science and Mathematics, New York University Abu Dhabi, PO Box 129188, Abu Dhabi, United Arab Emirates*
[3]*Department of Chemistry, New York University, New York, NY 10003, United States*
[4]*Smart Materials Lab, New York University Abu Dhabi, P.O. Box 129188, Abu Dhabi, United Arab Emirates*
[5]*Research Center for Environment and Materials, Macedonian Academy of Sciences and Arts, Bul. Krste Misirkov 2, MK–1000 Skopje, Macedonia*
[6]*Molecular Design Institute, Department of Chemistry, New York University, 100 Washington Square East, New York, NY 10003, United States*

*E-mail: durga.karothu@nyu.edu, serdal@nyu.edu, pance.naumov@nyu.edu

Supporting methods

Molecular modelling

Indentations were performed for 9,10-dibromoanthracene (DBA), theophylline anhydrate, L-alanine, L-threonine, and α-glycine on the planes (001), (100), (001), (001), and (001), respectively. All crystal systems were built using the respective CIFs obtained from the Cambridge Crystallographic Data Centre (CCDC).[1] Initial simulation cells were prepared by replicating the crystal unit cells along the **a**, **b**, and **c** axes such that the dimensions would be around 20 nm in each direction. Next, each simulation box was extended to 70 nm in the direction perpendicular to the indentation crystallographic planes to include the indenter and the template. An iron template with a thickness of 1.07 nm was positioned below the crystal to keep the bottom layers of the crystal fixed during indentation. This is achieved by applying position restraints to the template atoms during simulations. The system was first equilibrated under constant-volume, constant-temperature (NVT) conditions for 25 ps. Next, a spherical iron indenter was introduced with a radius of 3 nm. Although a pyramidal Berkovich indenter is usually used in the experiments, the shape would not have much effect in the simulations because this type of indenter has a rounded tip with up to 150 nm radius. A second NVT equilibration of 500 ps then followed to stabilize the system and bring the indenter into direct contact with the crystal surface. Both the indenter and the template were prepared using the nanomaterial modeler in CHARMM-GUI.[2]

The AMBER force field[3] was used to model the interactions of the organic crystals. The partial charges of the atoms of the organic molecules were calculated using the electrostatic potential module in NWChem.[4] The charges were calculated at the B3LYP/6-311g(d,p) level of theory.[5] Once the crystals were constructed, simulations were performed using GROMACS[6] software. Periodic boundary conditions were applied in all directions, with electrostatics treated by Particle-mesh Ewald (PME) method.[7] A cutoff distance of 1.2 nm was used for the van der Waals and electrostatic interactions. The equations of motion are solved with 1 fs time steps using leap-frog integrator.[8] For each simulation setup, a consecutive minimization and an NVT equilibration for 25–500 ps at T = 298.15 K was employed before the loading.

Nanoindentation simulations protocol

To apply an external force to the indenter, the PLUMED[9] plugging was used. Indentation simulations were carried out in the NVT ensemble with Berendsen temperature coupling[10] at T = 298.15 K and at a constant penetration rate, which is 8.3 m s^{-1}. Then, an equilibration step was employed where the dislocations were allowed to relax for 1 ns. After that, the indenter was retracted until the forces became zero. The latter stage is conducted at different unloading rates: (0.8, 0.4, 0.08, 0.008 m s^{-1}) to study the role of loading rate on Young's modulus (E). Atomic-scale deformation parameters were analyzed using the Open Visualization Tool (OVITO) software package.[11]

Computing Young's modulus from MD simulations

Force-displacement (*F*-*h*) curves from the steered MD simulation plotted against the indenter displacement (*h*). The data was fitted to the Oliver–Pharr (O–P) method[12,13] to determine *E*. Since it is assumed that the elastic deformation is recovered in the initial stage of unloading, the slope of the first part of the curve is used to determine the elastic modulus. The unloading curve is then fitted according to the power law relation:

$$F = a\,(h - b)^c \qquad (1)$$

where a, b, c are law fitting constants and *h* is the displacement.

Then, the elastic unloading stiffness, *S*, is calculated as the slope (Fig 1 (d)) from the upper portion of the unloading curve, and the stiffness is estimated as: $S = \frac{dF}{dh}$

Two other parameters must be computed to determine the *E*, which are:

$$h_c = h_{max} - \epsilon\,(F_{max}/S) \qquad (2)$$

where h_c is the actual contact depth, h_{max} and F_{max} are the maximum indentation depth and the measured force, respectively. ϵ is a constant that depends on the shape of the indenter and is taken as 0.75 for a spherical indenter[12,13].

The second parameter necessary to compute, *E*, is the indenter contact area A_c given in equation (3):

$$A_c = \pi\,[\,(2r_s h_c - {h_c}^2)^{\frac{1}{2}} + L_{max}]^2 \qquad (3)$$

where r_s is the indenter radius and L_{max} is a correction value for the indenter-crystal contact that is assumed to be equal to a typical bond length (0.2 nm).

Having the A_c and S values, the effective elastic modulus E_{eff} is calculated as:

$$E_{eff} = \frac{S\sqrt{\pi}}{2\sqrt{A_c}} \qquad (4)$$

from E_{eff} , the Young's modulus E is estimated as:

$$\frac{1-\nu^2}{E} = \frac{1}{E_{eff}} + \frac{1-\nu_i{}^2}{E_i} \qquad (5)$$

ν is the Poisson's ratio of the organic crystal; E_i and ν_i are the indenter elastic modulus and Poisson's ratio, which are neglected because the indenter is assumed to be a rigid sphere with relatively high E_i. Consistent with experiments, we used ν = 0.3.

Supporting files

A detailed description of the procedure used to calculate the Young's modulus for each crystal is provided in this section. All relevant files are included in the zipped folder named *Supporting_files.zip*, which contains two subfolders: *MD-nanoindentation* and *DFT*.

The *MD-nanoindentation* subfolder includes the input and output files required for performing the nanoindentation simulations. For each crystal, it contains the initial configuration file (*_initial.gro*) used to start the simulation, the final structure file (*_final.gro*) obtained after indentation, as well as the topology (*.top*) and parameter (*.mdp*) files used during the indentation simulations. The topology file defines the molecular structure, atom types, and interaction force fields needed for the simulation. Details about the parameters used in the *mdp* file are described above in the Supporting methods section. A *plumed_md.dat* file is also included, which defines the steered molecular dynamics protocol used for nanoindentation.[9] It defines the indentation setup—including the direction of indentation, the applied force on the indenter, and the displacement of the indenter relative to the crystal surface. It also tracks the indenter's center-of-mass displacement along the indentation direction, and applies a time-dependent moving harmonic restraint to simulate loading, equilibration, and unloading phases. Key simulation observables—such as the indenter's position and the force applied are recorded in the *COLVAR* output file every 0.01 ps for post-analysis. These data are used to plot the force-displacement curves from which Young's modulus is calculated. A python script (*calculation_of_E.py*) was used in order to fit the unloading curve according to Oliver–Pharr (O–P)[12,13] method as detailed in the Computing Young's Modulus from MD simulations section. The script calculates the slope of the upper linear part of the unloading curve and eventually computes E for each crystal. The resulting values of E are reported in Figure 1f.

The *DFT* subfolder contains the input and output files related to the density functional theory (DFT) optimization of the dibromoanthracene and theophylline anhydrate crystals. The *param* file describes the DFT settings used for the optimization calculations, as detailed in Density Functional Theory (DFT) Calculation of the Elastic Tensors subsection of the Computational Methodology in the main text. The optimized structures, obtained from *.castep* output files, were used as an input in Materials Studio[14] to generate a series of distorted structures required for calculating the full elastic tensor. The resulting Young's modulus values, derived from these elastic constants, were then compared to our nanoindentation E values, as shown in Figure 1h.

Supporting figure

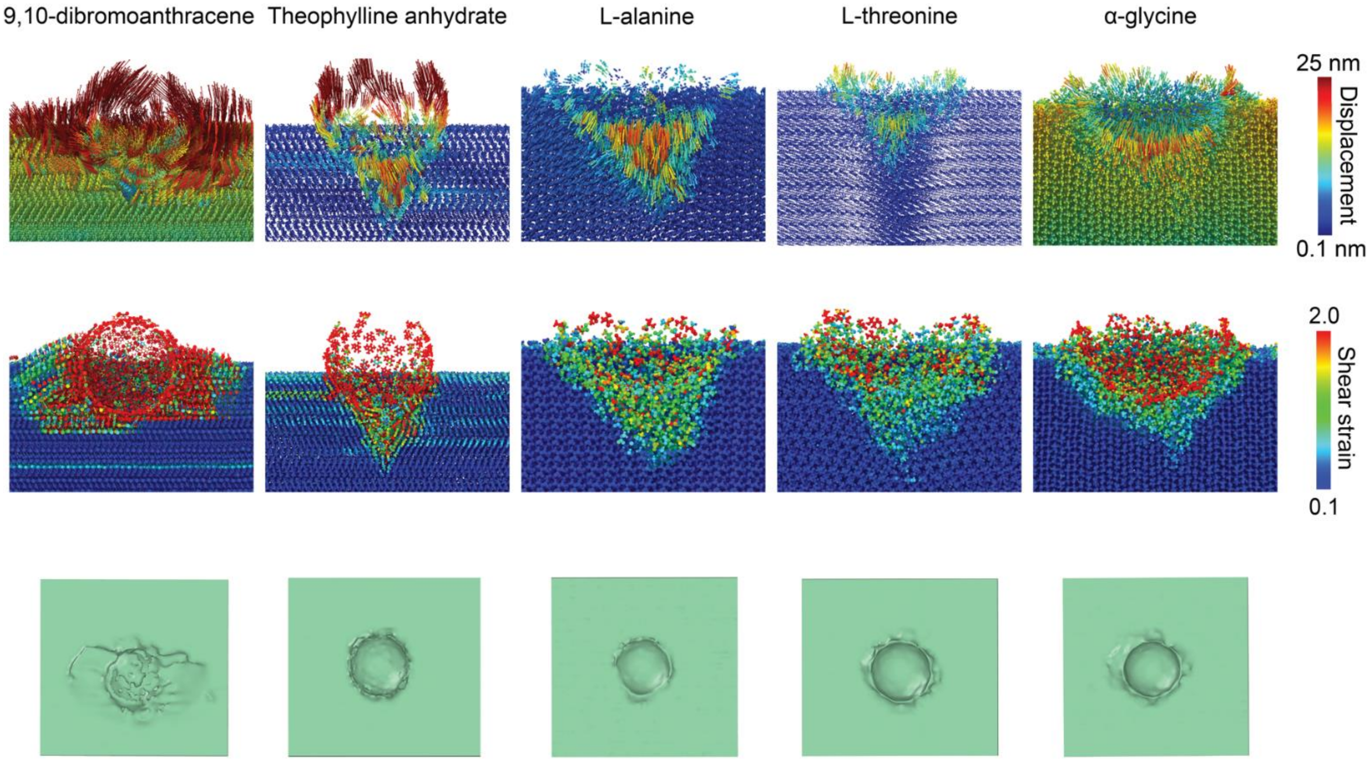

Figure S1. Displacement vectors, shear strain, and surface mesh of organic crystals after unloading.

Supporting references

(1) Groom, C. R.; Bruno, I. J.; Lightfoot, M. P.; Ward, S. C. The Cambridge Structural Database. *Acta Crystallogr. Sect. B* **2016**, *72*, 171–179. https://doi.org/10.1107/S2052520616003954.

(2) Jo, S.; Kim, T.; Iyer, V. G.; Im, W. CHARMM-GUI: A Web-Based Graphical User Interface for CHARMM. *J. Comput. Chem.* **2008**, *29*, 1859–1865. https://doi.org/10.1002/jcc.20945.

(3) Wang, J.; Wolf, R. M.; Caldwell, J. W.; Kollman, P. A.; Case, D. A. Development and Testing of a General Amber Force Field. *J. Comput. Chem.* **2004**, *25*, 1157–1174. https://doi.org/10.1002/jcc.20035.

(4) Valiev, M.; Bylaska, E. J.; Govind, N.; Kowalski, K.; Straatsma, T. P.; Van Dam, H. J. J.; Wang, D.; Nieplocha, J.; Apra, E.; Windus, T. L.; de Jong, W. A. NWChem: A Comprehensive and Scalable Open-Source Solution for Large Scale Molecular Simulations. *Comput. Phys. Commun.* **2010**, *181*, 1477–1489. https://doi.org/10.1016/j.cpc.2010.04.018.

(5) Becke, A. D. Density-functional Thermochemistry. III. The Role of Exact Exchange. *J. Chem. Phys.* **1993**, *98*, 5648–5652. https://doi.org/10.1063/1.464913.

(6) Abraham, M. J.; Murtola, T.; Schulz, R.; Páll, S.; Smith, J. C.; Hess, B.; Lindahl, E. GROMACS: High Performance Molecular Simulations through Multi-Level Parallelism from Laptops to Supercomputers. *SoftwareX* **2015**, *1–2*, 19–25. https://doi.org/10.1016/j.softx.2015.06.001.

(7) Essmann, U.; Perera, L.; Berkowitz, M. L.; Darden, T.; Lee, H.; Pedersen, L. G. A Smooth Particle Mesh Ewald Method. *J. Chem. Phys.* **1995**, *103*, 8577–8593. https://doi.org/10.1063/1.470117.

(8) Hockney, R. W.; Goel, S. P.; Eastwood, J. W. Quiet High-Resolution Computer Models of a Plasma. *J. Comput. Phys.* **1974**, *14*, 148–158. https://doi.org/10.1016/0021-9991(74)90010-2.

(9) Tribello, G. A.; Bonomi, M.; Branduardi, D.; Camilloni, C.; Bussi, G. PLUMED 2: New Feathers for an Old Bird. *Comput. Phys. Commun.* **2014**, *185*, 604–613. https://doi.org/10.1016/j.cpc.2013.09.018.

(10) Berendsen, H. J. C.; Postma, J. P. M.; van Gunsteren, W. F.; DiNola, A.; Haak, J. R. Molecular Dynamics with Coupling to an External Bath. *J. Chem. Phys.* **1984**, *81*, 3684–3690. https://doi.org/10.1063/1.448118.

(11) Stukowski, A. Visualization and Analysis of Atomistic Simulation Data with OVITO–the Open Visualization Tool. *Model. Simul. Mater. Sci. Eng.* **2009**, *18*, 015012. https://doi.org/10.1088/0965-0393/18/1/015012.

(12) Oliver, W. C.; Pharr, G. M. An Improved Technique for Determining Hardness and Elastic Modulus Using Load and Displacement Sensing Indentation Experiments. *J. Mater. Res.* **1992**, *7*, 1564–1583. https://doi.org/10.1557/JMR.1992.1564.

(13) Oliver, W. C.; Pharr, G. M. Measurement of Hardness and Elastic Modulus by Instrumented Indentation: Advances in Understanding and Refinements to Methodology. *J. Mater. Res.* **2004**, *19*, 3–20. https://doi.org/10.1557/jmr.2

(14) Biovia, D. S. Materials Studio. *R2 Dassault Systèmes BIOVIA San Diego* **2017**.
